# AXL, along with PROS1, is overexpressed in papillary thyroid carcinoma and regulates its biological behaviour

**DOI:** 10.1186/s12957-022-02801-0

**Published:** 2022-10-06

**Authors:** Mingze Wei, Yizeng Wang, Yuanchao Liu, Dongyang Li, Xianghui He

**Affiliations:** 1 Department of General Surgery, Ningbo First Hospital, Ningbo Hospital of Zhejiang University, Zhejiang Province, Ningbo, China; 2 Department of General Surgery, Tianjin Medical University General Hospital, Tianjin, China

**Keywords:** PROS1, AXL, Papillary thyroid carcinoma, Biological behaviour

## Abstract

**Background:**

AXL, a TAM tyrosine kinase receptor, plays an essential role in the pathogenesis of various solid tumours. This study explores the role of AXL and its ligand PROS1 in the generation and biological behaviour of papillary thyroid cancer (PTC).

**Methods:**

The expression levels of AXL in PTC cancer tissue were analysed using immunohistochemistry (IHC) staining. The expression levels of AXL in PTC and normal thyroid cell lines were analysed using real-time quantitative polymerase chain reaction (RT-qPCR). CCK-8 was used to assess the proliferation of the PTC cell line with and without the effect of the AXL inhibitor (R428). Scratching assays played a role in evaluating the cell migration rate.

**Results:**

PROS1 and AXL were expressed in TPC-1, B-CPAP, and Nthy-Ori 3–1 cells at different levels. Expression was significantly higher in PTC cell lines (TPC-1 and B-CPAP) than in the normal thyroid cell line (Nthy-Ori 3–1) (*p* < 0.05). In addition, AXL expression in PTC tissues was significantly higher than in adjacent normal tissues (*p* < 0.05). CCK-8 experiments confirmed that R428 suppresses the proliferation of PTC cell lines in a dose-dependent manner, with an increase in concentration from 0.5 to 4 μM, decreasing the inhibitory effect (*p* < 0.01). In addition, R428 inhibited PTC cell line migration to different degrees in a range of concentrations from 0.5 to 2 μM compared to control cells (*p* < 0.01).

**Conclusion:**

PROS1 and its downstream receptor AXL expression were significantly higher in PTC than in normal thyroid cells. AXL expression was also higher in human PTC tissues than in normal thyroid tissues. Inhibiting the PROS1-AXL-mediated TAM signaling pathway via the AXL blocker R428 suppressed the proliferation and migration of human PTC cells, highlighting the role of this cascade in human PTC development and progression.

## Background

According to the American SEER epidemiological database, thyroid cancer is the most common malignancy of the endocrine system and accounts for 3% of all new cancer cases [1]. Its increasing incidence over the past 30 years is almost entirely attributable to the higher diagnosis rate for differentiated thyroid cancers (DTCs), particularly papillary thyroid carcinoma (PTC), while the incidence of follicular thyroid carcinoma (FTC), anaplastic thyroid carcinoma (ATC), and medullary thyroid carcinoma (MTC) has remained relatively stable [2–4].

TAM receptor tyrosine kinase (TAM-RTK) is the general name for three subfamilies of tyrosine kinases: TYRO3, AXL, and MERTK. These are primary innate immunosuppressive receptors, with their canonical ligands (GAS6 and PROS1) sharing high structural homology. TAM receptors are widely distributed throughout the circulatory [5], reproductive [6], endocrine, and immune systems [7] and are primarily expressed in antigen-presenting cells (APCs) and natural killer (NK) cells of the immune system [8]. Notably, the TAM-RTK family receptors, including AXL, bind the ligands GAS6 and PROS1 to trigger several downstream signaling cascades (including the MAPK, PI3-K/AKT1, JAK/STAT, and NF-κB pathways), stimulating cell proliferation and survival [9]. Phosphatidylserine (PtdSer) is a major coregulator of the TAM signaling pathway, binding PROS1 or GAS6 ligands, and is implicated in tumour immunity and inflammation [10]. Furthermore, TYRO3, AXL, MERTK, and their corresponding ligands are involved in independent signaling pathways, with PROS1/AXL signaling pathway-mediated tumour proliferation being dominant in oral squamous cell carcinoma, whereas the other two TAM receptors (MERTK and TYRO3) are nearly undetectable.

TAM-RTK plays a major rate-limiting role in innate immune cell function, conferring APC tolerance and ultimately inhibiting antitumour cytotoxic T cells. TAM-driven tumour-promoting pathways in M2 polarization, including the inhibition of DC activation and the suppression of chemokines that mediate T-cell recruitment, all lead to the immune escape of malignant tumours. Therefore, therapies targeting TAM innate immune checkpoints hold great promise. Notably, BGB324 (also known as R428), a small molecule inhibitor of AXL, has exhibited significant inhibitory effects on tumour proliferation and migration in various cell lines, including squamous cell carcinoma, head and neck cancer, and breast cancer cells. Breast cancer xenograft experiments in mice also have demonstrated that R428 could suppress tumour angiogenesis and inhibit tumour cell migration [11–14].

AXL has also been verified to be highly expressed in anaplastic and medullary thyroid carcinoma, and research referring to AXL-targeted inhibitors in anaplastic or medullary thyroid carcinoma has yielded clinical value [15, 16]. Moreover, several targeted therapies based on tyrosine kinase inhibitors (TKIs) have played a promising role in metastatic differentiated thyroid cancer, including radioactive iodine-refractory (RAIR) papillary thyroid carcinoma [17–19]. Having identified AXL as a tyrosine kinase family with similar attributes, we aimed to explore the PROS1-AXL-mediated TAM signaling pathway for the clinical management and prognosis of papillary thyroid cancer by providing a deeper understanding of its underlying molecular mechanisms.

## Materials and methods

### Clinical samples

Samples were collected from 40 patients who underwent surgical treatment with postoperative preparation of paraffin sections, followed by PTC diagnosis by the Department of Thyroid, Breast, and Hernia Surgery, Tianjin Medical University General Hospital. Of these patients, seven were males, and 33 were females. The age range was 22–73 years, with an average age of 42.18 ± 12.50. There were 16 cases of PTC and 24 cases of PTMC among the 40 patients. In addition, 40 cancer tissue samples and 40 adjacent normal thyroid tissues were collected. The clinical data of the cases were complete, and all postoperative pathological diagnoses were confirmed. Patients with primary hyperthyroidism, recurrent thyroid cancer, other malignant cancers, systemic immune diseases, and infectious diseases were excluded.

### Immunohistochemistry

Paraffin-embedded tissue blocks from PTC patients who underwent thyroid lobectomy and specimens were retrieved from the pathology collection and sectioned to a thickness of 5 µm. The PTC tissue was designated the experimental group (40 individuals), while the adjacent normal thyroid (40 individuals) was designated the control group. All tissues were subjected to immunohistochemistry (IHC). The tissues were stained with an anti-AXL antibody (R&D Systems, USA) and routine HE. AXL expression was semi quantitated using the semiquantitative immunoreactive scoring system (IRS) as described previously [20], and the expression was defined as negative (*IRS* ≤ 1), mild positive (2 ≤ *IRS* ≤ 3), or strong positive (*IRS* ≥ 4) according to the percentage and intensity scores. All tissues were independently assessed by two pathologists who were blinded to the clinical data.

### Cell culture

RPMI-1640 (Solarbio, Beijing), high-glucose DMEM (HyClone, USA), and F12K media (Gibco, USA) were prepared with 10% (v/v) foetal bovine serum (FBS) and with 100 U/mL penicillin, 0.1 mg/mL streptomycin, and 2 mM L-glutamine (Gibco, USA) and used to culture the TPC-1, B-CPAP, and Nthy-Ori 3–1-cell lines, respectively. Cells were kept at 37 °C and 5% CO_2_. Every 2 days, the cell cultures were checked for microorganism contamination and the growth medium replaced. Cells were regularly passaged and frozen based on their growth and density. The TPC-1 and B-CPAP cell lines were derived from human PTC cancer tissue, while the Nthy-Ori 3–1-cell line was derived from normal human thyroid tissue. The CCK-8 kit and the AXL inhibitor R428 were purchased from MCE (USA).

### RT-qPCR

Cells were harvested and washed once with PBS. RNA was isolated using RZ lysis buffer (Tiangen Biotech, Beijing). cDNA was synthesized using the Fast Quant cDNA first-strand synthesis kit (Tiangen Biotech, Beijing). The 2 × SuperReal PreMix Plus kit (Tiangen Biotech, Beijing) was used for the RT-PCR system (20 μL volume). The amplification reaction was performed on a DNA Engine Opticon 2 real-time fluorescent quantitative PCR instrument (Bio-Rad, USA) per the manufacturer’s instructions. β-actin was employed as a housekeeping gene, and the *ΔΔ* threshold cycle (Ct) method was used. RT-qPCR results with *Ct* < 35, no specific peaks in the melting curve, and typical S-shaped amplification curves were included for standard calculations.

Primers were obtained from Beijing AuGCT DNA-SYN Biotechnology Co., Ltd. and are listed below.

**Table:** 

**Internal reference gene**	Primer sequence
β-actin	Forward 5′-3′: GCGAGAAGATGACCCAGCTC
	Reverse 5′-3′: CCAGTGGTACGGCCAGAGG
**Target genes**	
PROS1	Forward 5′-3′: TTGCACTTGTAAACCAGGTTGG
	Reverse 5′-3′: CAGGAACAGTGGTAACTTCCAG
AXL	Forward 5′-3′: TGGGTGAGGATGAACAGGATG
	Reverse 5′-3′: CTTCGCAGGAGAAAGAGGATG

### Cell proliferation experiment

TPC-1 and B-CPAP PTC cells in the logarithmic growth phase were collected. The number of cells in the prepared cell suspension was counted using a cell counter, and TPC-1 and B-CPAP cells were then inoculated. Cell culture medium was sequentially used to dilute cells based on a defined ratio to produce a gradient of different cell concentrations. Five cell concentration gradients were produced with six replicate wells per group. The outer perimeter of wells in the 96-well plate was not used for experiments due to edge effects. After inoculation, the cells were cultured for 12 h until they adhered to the plate surface. Ten microlitres of CCK-8 reagent was added per 100 μL medium, and the OD absorbance at 450 nm was measured after 0.5 h, 1 h, and 2 h using a microplate reader. The group with the OD closest to 1 and the best repeatability was determined to have the optimal concentration for cell inoculation in subsequent experiments.

### R428 inhibited cell proliferation

The experimental groups comprised TPC-1 cells and B-CPAP cells treated with R428, while the control group comprised untreated cells. Cell suspensions (100 μL/well) were inoculated in a 96-well plate at a cell concentration of 1.6 × 10^4^/well. Culture plates were placed in an incubator and precultured for 12 h until the cells adhered. Different concentrations of the AXL inhibitor R428 were added to the culture plate, and the exact same amount of PBS was added to the control group. After the R428 addition, the culture plate was placed in an incubator and incubated for 24 h. Ten microlitres of CCK-8 solution was carefully added to each well using a pipette, and the wall of the well was used as much as possible to prevent air bubbles. The culture plate was placed in an incubator and incubated for 2 h. The OD absorbance at 450 nm was measured using a microplate reader, and then, cell viability was calculated using the formula:

$$\mathrm{cell}\;\mathrm{survival}\;\mathrm{rate}=\;\frac{(As-Ab)}{(Ac-Ab)}\;\times\;100\%$$

- As: Sample well absorbance (containing cells, cell culture medium, CCK-8 solution, and R428 solution)
- Ac: Control well absorbance (containing cells, cell culture medium, and CCK-8 solution; no R428).
- Ab: Blank well absorbance (containing cell culture medium and CCK-8 solution; no cells or R428).

The experiment was performed in triplicate, and the average values are presented.

### Scratch assay

The experimental groups comprised TPC-1 cells and B-CPAP cells treated with R428, while the control group comprised untreated cells. First, evenly spaced horizontal lines were drawn every 0.5–1 cm across each well on the back of a 6-well plate using a marker and a ruler. At least three lines were drawn across each well. The type of cell to be added to each well was determined beforehand. Each well was inoculated with approximately 5 × 10^5^ cells. The specific number of cells varied among cell lines, but the general aim was to grow the cells to confluence overnight. The next day, a pipette tip was drawn along the horizontal line, forming a scratch amidst the cells. The pipette tip was held vertically and not diagonally. The cells were washed three times with PBS to remove scratched cells, and a low-concentration FBS (1%) culture medium was added to eliminate the effects of cell proliferation under normal serum concentrations on cell migration. Different concentrations of R428 were added to the experimental group, with the same volume of low-concentration FBS (1%) was added to the control group. Cells were incubated at 37 °C and 5% CO_2_. Samples were collected at 0 and 24 h and imaged.

## Statistics

For AXL, relative expression data in PTC and cancer-adjacent tissues were analysed using a *t*-test or one-way ANOVA and the Mann‒Whitney *U*- or Kruskal‒Wallis *H*-test, in SPSS 25.0. The correlation between the intensity of AXL expression in PTC tissues and clinicopathological characteristics was evaluated using Kruskal‒Wallis *H*-tests and one-way ANOVA in SPSS 25.0. The *T*-tests, Mann‒Whitney *U*-tests, one-way ANOVA, and Kruskal‒Wallis *H*-tests were used to assess the relative expression of AXL in various cell lines, cell proliferation, and cell migration under the AXL inhibitor R428 effect. GraphPad Prism 7.0 software was used for statistical analysis and graph preparation. Differences with *p* < 0.05 were considered statistically significant.

## Results

### AXL is highly expressed in PTC tissue

**Fig. 1:**
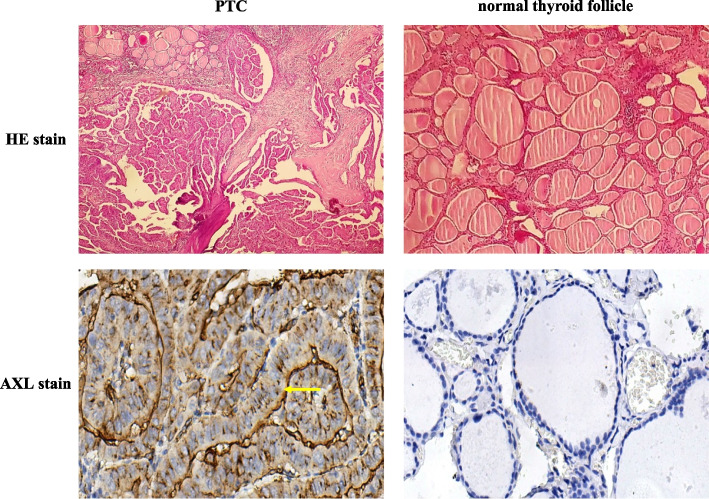
AXL expression in PTC and normal thyroid tissue. Paraffin-embedded slides were collected from 40 PTC tissues and 40 adjacent normal thyroid tissues. The PTC tissue was considered the experimental group, while the adjacent normal thyroid was considered the control group. Both groups were treated using immunohistochemical staining with AXL antibody and routine haematoxylin–eosin (HE) staining. The results indicate that AXL is highly expressed in PTC tissue (yellow arrow)

In all AXL-positive specimens, staining was observed in the cell membrane and cytoplasm. However, some samples exhibited more significant staining in the cell membrane than in the cytoplasm and vice versa. It should be noted that, in addition to thyroid follicles, vascular endothelial cells, monocytes, and red blood cells also exhibited various degrees of staining, which was not considered part of the results [9]. Among thyroid cancer tissues, 32.5% of cases exhibited strong positive staining (13/40 cases), 45.0% exhibited weak positive staining (18/40 cases), and 22.5% exhibited negative staining (9/40 cases). In cancer-adjacent tissues, 0.0% of cases exhibited strong positive staining (0/40 cases), 55.0% exhibited weak positive staining (22/40 cases), and 45.0% exhibited negative staining (18/40 cases). Based on the Mann‒Whitney *U*-test, the difference between cancer and adjacent tissues was statistically significant (*p* < 0.05) (Table 1, Fig. 1A and B).

**Table 1:** AXL expression in PTC and cancer-adjacent tissues

Group	Strong positive	Weak positive	Negative	*p*-value
Cancer tissue	13	18	9	Mann–Whitney *U*
Cancer-adjacent tissue	0	22	18	0.001

### Correlation of AXL expression in PTC tissues with clinicopathological characteristics

AXL immunohistochemistry results from patient PTC tissues were compared to the patient clinicopathological data. The Kruskal‒Wallis *H*-tests and one-way ANOVA indicated that there were no significant differences in age, sex, tumour size, lymph node metastasis, TNM stage, or American Joint Committee on Cancer (AJCC) stages among the different intensities of positive AXL expression (*p* > 0.05) (Table 2).

**Table 2:** Correlation between intensity of AXL expression in PTC tissues and clinicopathological characteristics

Variable	Strong positive	Weak positive	Negative	*p*-value
Frequency (no. of patients)	13	18	9	
Mean age (years)	40.31 ± 13.76	44.72 ± 10.87	39.78 ± 14.15	0.332
Sex				0.305
M	1 (7.7)	5 (27.8)	1 (11.1)	
F	12 (92.3)	13 (72.2)	8 (88.9)	
Microcarcinoma				0.157
Yes	5 (38.5)	13 (72.2)	6 (66.7)	
No	8 (61.5)	5 (27.8)	3 (33.3)	
Extrathyroidal extension				0.292
Yes	12 (92.3)	13 (72.2)	6 (66.7)	
No	1 (7.7)	5 (27.8)	3 (33.3)	
Tumour size				0.535
T1	1 (7.7)	4 (22.2)	2 (22.2)	
T2	0 (0)	0 (0)	0 (0)	
T3	12 (92.3)	14 (77.8)	7 (77.8)	
T4	0 (0)	0 (0)	0 (0)	
Lymph node involvement				0.253
N0	3 (23.1)	7 (38.9)	1 (11.1)	
N1a	9 (69.2)	8 (44.4)	5 (55.6)	
N1b	1 (7.7)	3 (16.7)	3 (33.3)	
Distant metastasis				
M0	0	0	0	
M1	0	0	0	
AJCC stage				0.735
I	8 (61.5)	8 (44.4)	5 (55.6)	
II	0 (0)	0 (0)	0 (0)	
III	4 (30.8)	9 (50.0)	3 (33.3)	
IV	1 (7.7)	1 (5.6)	1 (11.1)	

### Both AXL and its ligand PROS1 are overexpressed in the PTC cell line

**Fig. 2:**
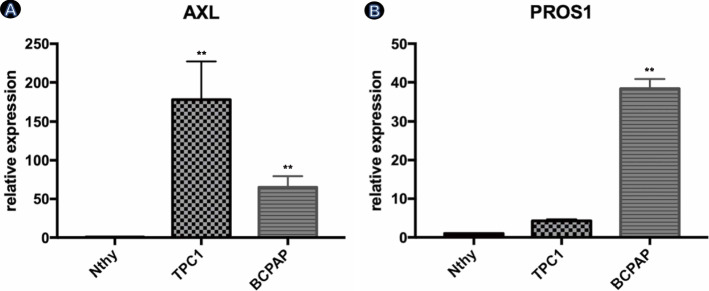
Relative expression of AXL and its ligand PROS1 in different cell lines. RNA was extracted from cultured *Nthy*, *TPC1*, and *BCPAP* cell lines separately. *TPC1* and *BCPAP* are *PTC* cell lines that have been classified into the experimental group, while Nthy is the control group. The results indicate that PROS1 and AXL mRNA relative expression in TPC1 and BCPAP cell lines are overwhelmingly higher than that in the Nthy cell line (**A**, **B**). (**p < 0.01 compared to the control group)

To further confirm the expression of PROS1-AXL pathway factors in PTC, we determined PROS1 and AXL expression in the TPC-1, B-CPAP, and Nthy-Ori 3–1-cell lines via RT-qPCR (Tables 3 and 4). The expression of PROS1 and AXL in the PTC cell lines TPC-1 and B-CPAP was significantly higher than in the normal thyroid cell line Nthy-Ori 3–1 (Fig. 2A and B). Data in the table are presented as the mean ± standard deviation. The GraphPad Prism 7.0 software was used for statistical analysis and graph preparation.

**Table 3:** Relative AXL mRNA expression in each cell line

**Group**	**Ct value**	**Ct (β-actin)**	*Δ***Ct**	**2**^**−**ΔΔ**Ct**^
Nthy-Ori 3–1	19.37 ± 0.22	11.12 ± 0.06	8.26 ± 0.22	1
TPC-1	12.66 ± 0.44	11.84 ± 0.35	0.83 ± 0.44	177.84 ± 49.43**
B-CPAP	15.36 ± 0.32	13.10 ± 0.15	2.26 ± 0.21	65.07 ± 14.35**

*Note*: Nthy-Ori 3–1 was the control group, **p < 0.01, *p < 0.05)

**Table 4:** Relative PROS1 mRNA expression in each cell line

**Group**	**Ct value**	**Ct (β-actin)**	*Δ***Ct**	**2**^**−**ΔΔ**Ct**^
Nthy-Ori 3–1	20.29 ± 0.54	13.31 ± 0.26	6.98 ± 0.54	1
TPC-1	19.14 ± 0.14	14.26 ± 0.58	4.88 ± 0.14	4.29 ± 0.41*
B-CPAP	16.30 ± 0.10	14.57 ± 0.34	1.72 ± 0.10	38.37 ± 2.50**

*Note*: Nthy-Ori 3–1 was the control group, **p < 0.01, *p < 0.05)

### Effect of the AXL inhibitor R428 on suppressing PTC cell proliferation

**Fig. 3:**
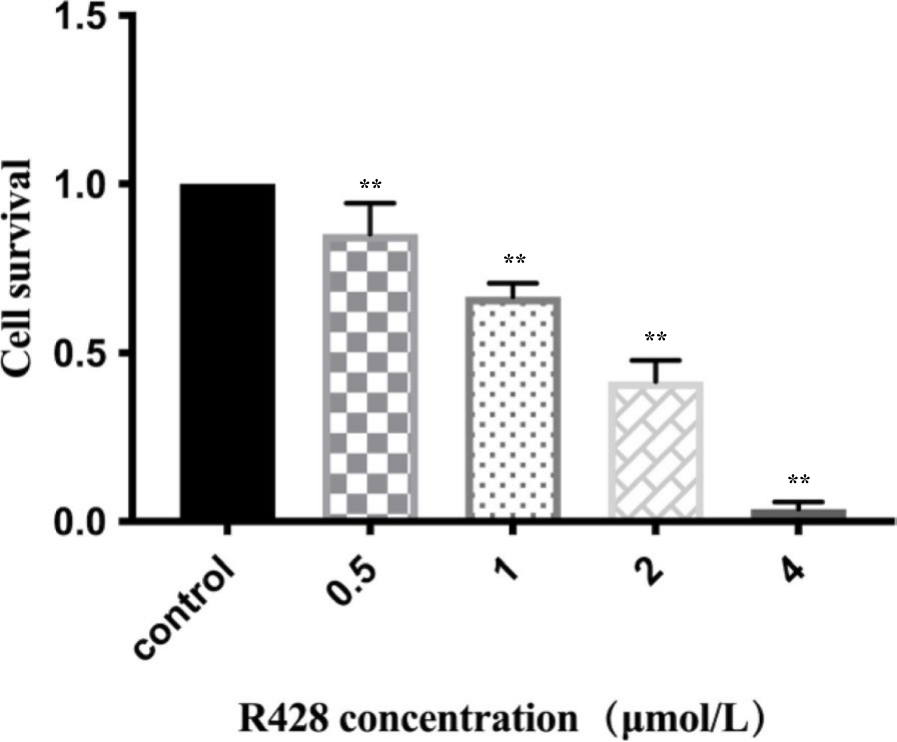
Effect of different concentrations of R428 on *B-CPAP* cell proliferation. The *B-CPAP* cell line was cultured and treated with the AXL inhibitor R428 at different concentrations (0.5 µmol/L, 1 µmol/L, 2 µmol/L, 4 µmol/L), and the untreated *B-CPAP* cell line was used as a control group. A CCK-8 assay was performed to assess the cell proliferation rate. The data shows that *B-CPAP* proliferation was suppressed by various concentrations of R428 (**p < 0.01 compared to the control group)

In the B-CPAP experimental group, the cell survival rates after treatment with 0.5, 1.0, 2.0, and 4.0 μmol/L R428 were 85.28% ± 9.21%, 66.74% ± 3.88%, 41.51% ± 6.33%, and 3.71% ± 2.10%, respectively. Further comparison of the cell survival rate between experimental groups treated with different concentrations of R428 indicated significant differences (*p* < 0.01). In addition, the inhibitory effect of R428 was stronger at higher concentrations, indicative of a concentration-dependent effect of R428 on B-CPAP cell proliferation (Fig. 3).

### Effect of R428 on the migration of PTC cells

**Fig. 4:**
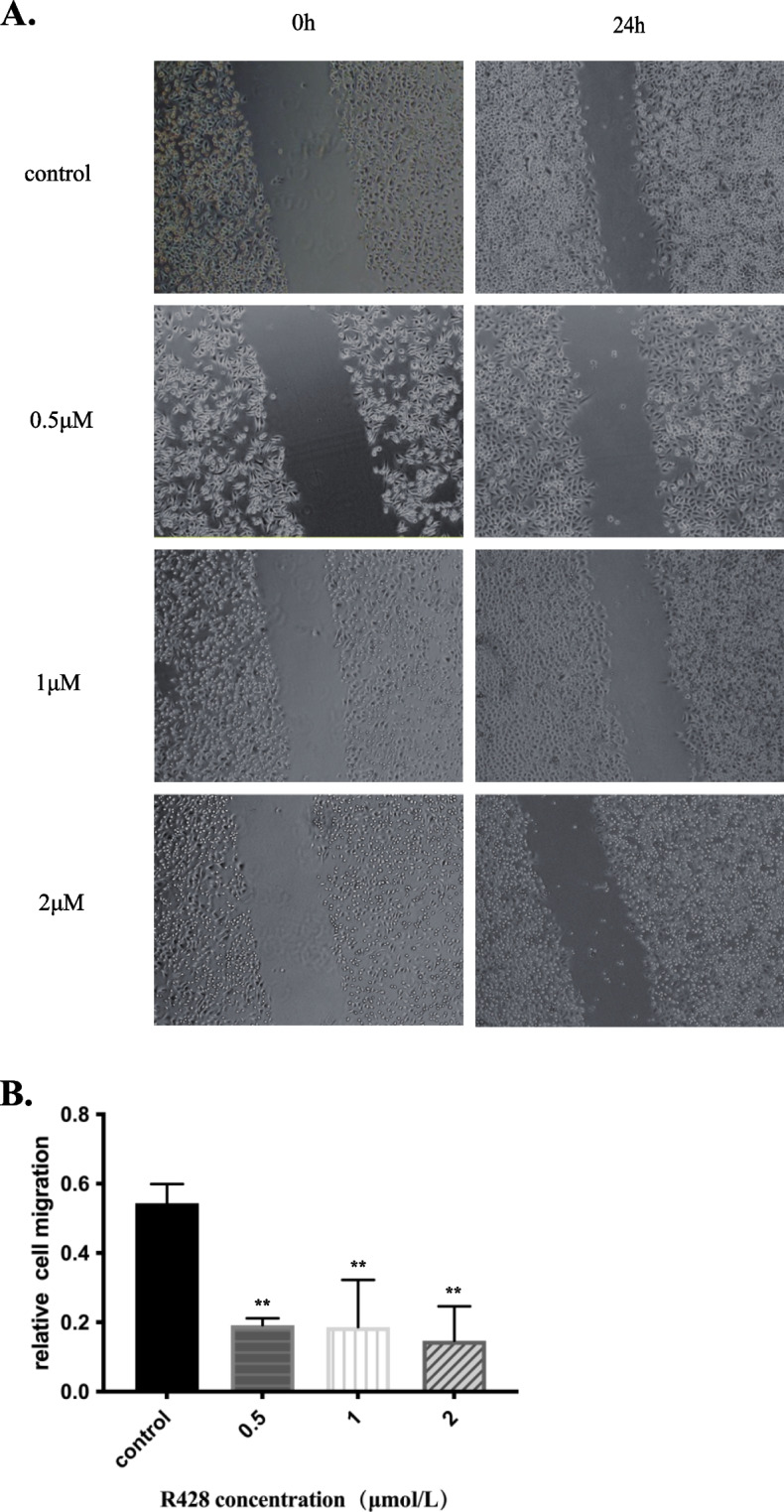
Effect of different concentrations of R428 on *B-CPAP* cell migration. *The B-CPAP cell line* was cultured and treated with the AXL inhibitor R428 at various concentrations (0.5 µmol/L, 1 µmol/L, 2 µmol/L), and the no R428-treated group was used as the control group. The scratch assay was used to evaluate cell migration in each group. The graph taken by inverted microscopy indicates that the scratching area of the experimental groups treated with R428 was not decreased compared with that of the control group (**A**). Data were processed using *ImageJ* software, and the cell migration rate was finally quantified. The column chart further illuminated that there were significant differences among the serial experimental groups and the control group (**B**). (**p < 0.01 compared to the control group)

Cells were divided into experimental groups (0.5 μmol/L, 1.0 μmol/L, and 2.0 μmol/L R428) and a control group (no. R428), and the migration ability of the cells was determined in each group. Imaging sample results were obtained using an inverted microscope at 100 × magnification, followed by processing with ImageJ software. Only a small number of cells migrated to the scratched area in the experimental group 24 h after scratching. In contrast, many cells migrated to the scratched area in the control group, significantly reducing the scratched area (Fig. 4A). The relative migration rates of the control group, 0.5 μmol/L R428 experimental group, 1.0 μmol/L R428 experimental group, and 2.0 μmol/L R428 experimental group were 54.35% ± 5.55%, 19.17% ± 1.98%, 18.62% ± 13.59%, and 14.67% ± 9.97%, respectively. Based on these results, the migration of the B-CPAP cells in each experimental group differed significantly from that of the control group (*p* < 0.01) (Fig. 4B).

## Discussion

PTC is the most common pathological subtype of thyroid cancer, particularly among DTC [21]. As most PTC patients have favourable clinical prognoses, investigating AXL expression in PTC tissues is of clinical relevance. The three TAM receptors, including AXL, have a similar overall domain composition, with an extracellular domain composed of two tandem immunoglobulin-like common structural domains (Ig1 and Ig2) and two tandem fibronectin type 3 (FN III) domains, which are primarily responsible for the binding of PROS1 or GAS6 ligands. In contrast, the intracellular domain is composed of a tyrosine kinase domain primarily responsible for mediating intracellular signaling, including the PI3K/AKT, ERK/MAPK, and JAK/STAT pathways. The ligands PROS1 and GAS6 also share domains and an overall 44% amino acid sequence homology [8].

In the present study, we investigated the expression of AXL in PTC cancer tissue and control tissue via immunohistochemical staining. AXL protein expression was significantly higher in PTC than in normal thyroid tissue, and positive staining was primarily observed in tumour cell membranes and cytoplasm. Further comparison of different AXL staining intensities with the clinicopathological characteristics of PTC patients revealed no significant differences based on age, sex, tumour size, or clinical stage. Xing M. et al. previously reported that mutations in the BRAF gene are associated with the development, lymph node metastasis, and clinical stage of PTC [22]. Xie et al. proposed that BRAF mutations are associated with pathological TNM stage, monocytes, systemic inflammatory response index (SIRI), and galectin-3 expression. SIRI is a major risk factor for BRAF mutations, as patients with a low SIRI have higher rates of mutated BRAF [23]. Recent studies have also shown that the intensity of positive AXL staining in the cancer tissues of PTC patients is positively correlated with radioactive iodine-refractory thyroid cancer, disease recurrence, and poor prognosis [9]. Therefore, taken together with our previously reported immunohistochemical analysis of PROS1, the TAM signaling pathway mediated via PROS1-AXL is upregulated in PTC tissues, with AXL playing a major regulatory role in PTC development and progression. Nevertheless, its effect on malignancy and prognosis remains to be further investigated. As the body of research on the BRAF^V600E^ gene mutation and the pathogenic mechanism concerning PTC prognosis expands, the investigation of similar genetic changes, such as AXL/PROS1 and RET/PTC rearrangement, represents another promising field in the study of PTC.

In the second part of the study, the expression and regulation of the TAM signaling pathway, as a major innate immune checkpoint signaling pathway and another TKI family in PTC, were further investigated. In recent years, as a member of the TAM family, AXL has received considerable attention in studies of tumour immunity. Studies have shown that AXL-mediated signaling can phosphorylate and activate the downstream ERK and AKT1 pathways [24, 25]. In addition, the downstream NF-κB pathway activated by GAS6/AXL/AKT1 inhibits the apoptosis of PTC cells [26], contributing to poor PTC prognosis. Many studies have reported that AXL is involved in the pathogenesis and biological behaviour of oral squamous cell carcinoma, gallbladder cancer, prostate cancer, and other cancers [27–29]. Most of these studies have focused on the role of the GAS6-AXL pathway, while there have been relatively few studies on PROS1, a primary upstream ligand of the AXL receptor. AXL has a similar structural component to the other two TAM receptors. In the formal part of this study, we preliminarily demonstrated that the PROS1 and AXL proteins are significantly overexpressed in PTC cancer tissues compared to normal thyroid tissues, but their expression was not associated with patient clinicopathological characteristics. In this section, we focus on exploring the expression of the PROS1-AXL pathway in PTC cell lines at the cellular and molecular levels and more importantly reveal its impact on biological functions.

The present study demonstrates that PROS1 and AXL mRNA expression is higher in PTC than in normal thyroid cells. When comparing two groups of PTC cell lines, AXL was more strongly expressed in TPC-1 cells, and PROS1 was more strongly expressed in B-CPAP cells. These results suggest the following hypotheses. First, as AXL has a different binding affinity for its two ligands, GAS6 and PROS1, in different PTC cell lines, it is possible that AXL preferentially binds to GAS6 in TPC-1 cells. As a result, the TAM signaling pathway mediated by GAS6-AXL regulates the tumour microenvironment and biological behaviour of this cell line. Furthermore, the TAM signaling pathway is widely active in various body tissues, including in tumour cells that upregulate TAM signaling through autocrine and paracrine PROS1 or GAS6, thereby facilitating a more favourable environment for tumour growth. Therefore, these results suggest the possibility that the proportion of ligands secreted by different PTC cell lines is the cause of the difference in expression between the groups. However, by comparing the experimental and control groups, this study demonstrates high PROS1-AXL expression in PTC cell lines at the mRNA level, supporting an important role for the PROS1-AXL-mediated TAM signaling pathway in the development and progression of PTC. Furthermore, compared to the untreated control group, the experimental groups of B-CPAP cells treated with R428 exhibited significantly lower proliferation in a concentration-dependent manner. These results demonstrate that blockade of AXL suppresses the proliferation of PTC, further supporting a major role for PROS1-AXL in PTC pathophysiology.

The TAM pathway has multiple tumour-promoting effects within the tumour microenvironment, including M2 polarization of macrophages, inhibition of DC activation, and suppression of chemokine production by T cells. Therefore, the specific signaling through which the PROS1-AXL pathway participates in PTC regulation remains to be investigated further. Nevertheless, the present study establishes a theoretical foundation for further investigation. PtdSer has also attracted considerable attention in recent years as a crucial costimulatory factor for the TAM pathway, directly participating in PROS1-AXL-mediated innate immunity and tumour microenvironment regulation [10]. For example, during the TAM-PtdSer-mediated phagocytosis of ACs, PROS1/GAS6 on macrophages binds to PtdSer on ACs through its Gla domain. Calcium ions then reinforce this binding. PROS1 and GAS6 act as adapter molecules that bridge the TAM receptors on phagocytes through the carboxy terminus to activate downstream signaling pathways, which then allow macrophages to bind to PtdSer moieties on ACs, thus stimulating phagocytosis [10]. The role of PtdSer in PROS1-AXL-mediated TAM signaling in the context of PTC and other solid tumours warrants further investigation. Elvira et al. found that AXL and GAS6 inhibit the spontaneous apoptosis of cancer cells during thyroid cancer development, and that blocking either AXL or GAS6 greatly affected PTC cell survival and resistance to apoptosis [30]. In recent years, another study by Elvira et al. showed that the AXL-mediated TAM signaling pathway negatively affects the prognosis and overall survival rate of radioactive iodine-refractory PTC [9]. In a previous study on the role of PROS1 in developing resistance to androgen deprivation therapy, Peng et al. found that high PROS1 expression promoted the proliferation of prostate cancer cells and exacerbated resistance [31]. Poupak et al. reported that the chemotherapy agent cabozantinib, an AXL inhibitor, effectively improved patients’ progression-free survival (PFS), while iodine resistance was reported in phase 2 clinical trials in DTC patients [32]. Masamichi et al. showed that gilteritinib could effectively inhibit the proliferation of haematopoietic cells in an FLT3 mutant mouse model of acute myeloid leukaemia [33].

Although DTC patients are usually known for their excellent prognoses, RAIR or metastatic RAIR thyroid cancers still have poor prognoses and limitations. Certainly, there is an ongoing need for the development of agents that can be effective despite RAI refractoriness. Meng et al. verified that a novel small-molecule tyrosine kinase inhibitor (TKI), apatinib, could safely act as an antitumour rule in DTC by altering biological behaviour through the PI3K/Akt/mTOR signaling pathway. More importantly, the autophagy effects induced by apatinib could also provide a potential targeted therapy for RAIR PTC [19]. In addition, a first-in-class Wee-1 inhibitor, adavosertib, exerted a cytotoxic effect on FTC or BRAF^V600E^ PTC in vivo and in vitro. Moreover, adavosertib can strengthen the tumour growth suppression of dabrafenib and trametinib [18]. It is worth noting that a series of patients with BRAF^V600E^ RAIR metastatic PTC showed improvement in the progression-free or overall survival after treatment with a BRAF/MEK inhibitor [17]. Regarding our study, AXL, along with its downstream signaling pathway, shares a pathogenesis of tumour oncogenesis similar to that of other tyrosine kinase families. Inspired by current research trends and understanding, it is encouraging that our research targeting AXL along with its corresponding TAM signaling pathway may have broad prospects for exploitation in targeted therapy for different kinds of DTC, especially RAIR PTC.

## Conclusion

In this study, the expression levels of AXL/PROS1 were elevated in human papillary thyroid carcinoma. More importantly, the AXL inhibitor R428 effectively reduced the proliferation and migration of PTC cells to varying degrees, supporting the significance of PROS1-AXL signaling in PTC biological functions. Further research on the inhibition of TAM signaling holds great promise for the treatment of PTC patients with poor prognoses.

## Data Availability

The datasets used and analysed during the current study are available from the corresponding author upon reasonable request.

## Abbreviations

PTC: Papillary thyroid carcinoma
TAM-RTK: TAM receptor tyrosine kinase
DTC: Differentiated thyroid carcinoma
FTC: Follicular thyroid carcinoma
ATC: Anaplastic thyroid carcinoma
MTC: Medullary thyroid carcinoma
PTMC: Papillary thyroid micro-carcinoma
TCR: T-cell receptor
TSH: Thyroid-stimulating hormone
APC: Antigen-presenting cell
DC: Dendritic cell
NK: Nature killing
AC: Apoptotic cell
TLR: Toll-like receptor
Type 1 IFNs: Type 1 interferon
TIDC: Tumour-infiltrating dendritic cell
NSCLC: Non-small cell lung cancer
IHC: Immunohistochemistry
AJCC: American Joint Committee on Cancer
RT-qPCR: Real-time quantitative PCR
HT: Hashimoto’s thyroiditis
TRAb: Thyroid-stimulating hormone antibody
Tg: Thyroglobulin
TPO: Thyroid peroxidase
TPOAb: Thyroid peroxidase antibody
PtdSer: Phosphatidylserine
SIRI: Systemic inflammation response index
FNAC: Fine-needle aspiration cytology
PFS: Progression-free survival
HE: Haematoxylin–eosin
RAIR: Radioactive iodine refractory

## References

[1] Lamartina L, Grani G, Durante C, Borget I, Filetti S, Schlumberger M (2018). Follow-up of differentiated thyroid cancer - what should (and what should not) be done. Nat Rev Endocrinol.

[2] Filetti S, Durante C, Hartl D, Leboulleux S, Locati LD, Newbold K, Papotti MG, Berruti A, Committee EG (2019). Thyroid cancer: ESMO Clinical Practice Guidelines for diagnosis, treatment and follow-updagger. Ann Oncol.

[3] Davies L, Welch HG (2014). Current thyroid cancer trends in the United States. JAMA Otolaryngol Head Neck Surg.

[4] Seib CD, Sosa JA (2019). Evolving understanding of the epidemiology of thyroid cancer. Endocrinol Metab Clin North Am.

[5] Angelillo-Scherrer A, Burnier L, Flores N, Savi P, DeMol M, Schaeffer P, Herbert J-M, Lemke G, Goff SP, Matsushima GK (2005). Role of Gas6 receptors in platelet signaling during thrombus stabilization and implications for antithrombotic therapy. J Clin Investig.

[6] Ito T, Ito M, Naito S, Ohtsuru A, Nagayama Y, Kanematsu T, Yamashita S, Sekine I (1999). Expression of the Axl receptor tyrosine kinase in human thyroid carcinoma. Thyroid.

[7] Sandahl M, Hunter DM, Strunk KE, Earp HS, Cook RS (2010). Epithelial cell-directed efferocytosis in the post-partum mammary gland is necessary for tissue homeostasis and future lactation. BMC Dev Biol.

[8] Burstyn-Cohen T (2017). TAM receptor signaling in development. Int J Dev Bio.

[9] Collina F, La Sala L, Liotti F, Prevete N, La Mantia E, Chiofalo MG, Aquino G, Arenare L, Cantile M, Liguori G (2019). AXL is a novel predictive factor and therapeutic target for radioactive iodine refractory thyroid cancer. Cancers.

[10] Burstyn-Cohen T, Maimon A (2019). TAM receptors, phosphatidylserine, inflammation, and Cancer. Cell Commun Signal.

[11] Sadahiro H, Kang K-D, Gibson JT, Minata M, Yu H, Shi J, Chhipa R, Chen Z, Lu S, Simoni Y (2018). Activation of the receptor tyrosine kinase AXL regulates the immune microenvironment in glioblastoma. Can Res.

[12] Hector A, Montgomery EA, Karikari C, Canto M, Dunbar KB, Wang JS, Feldmann G, Hong SM, Haffner MC, Meeker AK (2010). The Axl receptor tyrosine kinase is an adverse prognostic factor and a therapeutic target in esophageal adenocarcinoma. Cancer Biol Ther.

[13] Holland SJ, Pan A, Franci C, Hu Y, Chang B, Li W, Duan M, Torneros A, Yu J, Heckrodt TJ (2010). R428, a selective small molecule inhibitor of Axl kinase, blocks tumor spread and prolongs survival in models of metastatic breast cancer. Can Res.

[14] Li Y, Ye X, Tan C, Hongo JA, Zha J, Liu J, Kallop D, Ludlam MJC, Pei L (2009). Axl as a potential therapeutic target in cancer: role of Axl in tumor growth, metastasis and angiogenesis. Oncogene.

[15] Garg M, Kanojia D, Mayakonda A, Ganesan TS, Sadhanandhan B, Suresh S, S S, Nagare RP, Said JW, Doan NB (2017). Selinexor (KPT-330) has antitumor activity against anaplastic thyroid carcinoma in vitro and in vivo and enhances sensitivity to doxorubicin. Sci Rep.

[16] Pishkari S, Hadavi R, Koochaki A, Razaviyan J, Paryan M, Hashemi M, Mohammadi-Yeganeh S (2021). Assessment of AXL and mTOR genes expression in medullary thyroid carcinoma (MTC) cell line in relation with over expression of miR-144 and miR-34a. Horm Mol Biol Clin Investig.

[17] Jafri S, Yaqub A (2021). Redifferentiation of BRAF V600E-mutated radioiodine refractory metastatic papillary thyroid cancer after treatment with dabrafenib and trametinib. Cureus.

[18] Lu YL, Wu MH, Lee YY, Chou TC, Wong RJ, Lin SF (2021). Efficacy and biomarker analysis of adavosertib in differentiated thyroid cancer. Cancers (Basel).

[19] Meng X, Wang H, Zhao J, Hu L, Zhi J, Wei S, Ruan X, Hou X, Li D, Zhang J (2020). Apatinib inhibits cell proliferation and induces autophagy in human papillary thyroid carcinoma via the PI3K/Akt/mTOR signaling pathway. Front Oncol.

[20] Lin CW, Lin CC, Lee PH, Lo GH, Hsieh PM, Koh KW, Lee CY, Chen YL, Dai CY, Huang JF (2017). The autophagy marker LC3 strongly predicts immediate mortality after surgical resection for hepatocellular carcinoma. Oncotarget.

[21] Zhu J, Wang X, Zhang X, Li P, Hou H (2015). Clinicopathological features of recurrent papillary thyroid cancer. Diagn Pathol.

[22] Xing M, Westra WH, Tufano RP, Cohen Y, Rosenbaum E, Rhoden KJ, Carson KA, Vasko V, Larin A, Tallini G (2005). BRAF mutation predicts a poorer clinical prognosis for papillary thyroid cancer. J Clin Endocrinol Metab.

[23] Xie H, Wei B, Shen H, Gao Y (2018). BRAF mutation in papillary thyroid carcinoma (PTC) and its association with clinicopathological features and systemic inflammation response index (SIRI). Am J Transl Res.

[24] Ruan G-X, Kazlauskas A (2012). Axl is essential for VEGF-A-dependent activation of PI3K/Akt. EMBO J.

[25] Avilla E, Guarino V, Visciano C, Liotti F, Svelto M, Krishnamoorthy G, Franco R, Melillo RM (2011). Activation of TYRO3/AXL tyrosine kinase receptors in thyroid cancer. Cancer Res.

[26] Hasanbasic I, Cuerquis J, Varnum B, Blostein MD (2004). Intracellular signaling pathways involved in Gas6-Axl-mediated survival of endothelial cells. AJP-Heart Circ Physiol.

[27] Abboud-Jarrous G, Priya S, Maimon A, Fischman S, Cohen-Elisha M, Czerninski R, Burstyn-Cohen T (2017). Protein S drives oral squamous cell carcinoma tumorigenicity through regulation of AXL. Oncotarget.

[28] Gay CM, Balaji K, Byers LA (2017). Giving AXL the axe: targeting AXL in human malignancy. Br J Cancer.

[29] Jd P, Gj V, Rg C, Vasconcellos JF (2013). The receptor tyrosine kinase Axl is an essential regulator of prostate cancer proliferation and tumor growth and represents a new therapeutic target. Oncogene.

[30] Avilla E, Guarino V, Visciano C, Liotti F, Svelto M, Krishnamoorthy G, Franco R, Melillo RM (2011). Activation of TYRO3/AXL tyrosine kinase receptors in thyroid cancer. Can Res.

[31] Ning P, Zhong J-G, Jiang F, Zhang Y (2016). Role of protein S in castration-resistant prostate cancer-like cells. Endocr Relat Cancer.

[32] Fallahi P, Ferrari SM, Bari FD (2015). Cabozantinib in thyroid cancer. Recent Pat Anti-Cancer Drug Discovery.

[33] Mori M, Kaneko N, Ueno Y, Yamada M, Tanaka R, Saito R, Shimada I, Mori K, Kuromitsu S (2017). Gilteritinib, a FLT3/AXL inhibitor, shows antileukemic activity in mouse models of FLT3 mutated acute myeloid leukemia. Invest New Drugs.

